# Females with Type 2 Diabetes Mellitus Are Prone to Diabetic Retinopathy: A Twelve-Province Cross-Sectional Study in China

**DOI:** 10.1155/2020/5814296

**Published:** 2020-04-21

**Authors:** Mei Li, Yina Wang, Zifeng Liu, Xixiang Tang, Panwei Mu, Ying Tan, Jing Wang, Bairun Lin, Juan Deng, Ruiping Peng, Rongyu Zhang, Zhihui He, Dongling Li, Yongjun Zhang, Caixian Yang, Yuan Li, Yuming Chen, Xun Liu, Yanming Chen

**Affiliations:** ^1^Department of Endocrinology & Metabolism, Guangdong Provincial Key Laboratory of Diabetology, The Third Affiliated Hospital of Sun Yat-sen University, Guangzhou 510630, China; ^2^VIP Medical Service Center, The Third Affiliated Hospital of Sun Yat-sen University, Guangzhou 510630, China; ^3^Clinical Data Center, The Third Affiliated Hospital of Sun Yat-sen University, Guangzhou 510630, China; ^4^Department of Endocrinology, The Third Affiliated Hospital of Sun Yat-sen University, Yuedong Hospital, Meizhou 514021, China; ^5^Department of Ophthalmology, The Third Affiliated Hospital of Sun Yat-sen University, Guangzhou 510630, China; ^6^Guangzhou Da'an Clinical Laboratory Center Co. Ltd., Guangzhou 440100, China; ^7^Guangdong Provincial Center for Disease Control and Prevention, Guangdong Provincial Institute of Public Health, Guangzhou 510000, China; ^8^Department of Endocrinology, The Fifth Affiliated Hospital of Zunyi Medical University, Zhuhai 519100, China; ^9^Department of Endocrinology & Metabolism, Qingyuan People's Hospital, Qingyuan 511518, China; ^10^School of Public Health, Sun Yat-sen University, Guangzhou 510080, China; ^11^Department of Nephrology, The Third Affiliated Hospital of Sun Yat-sen University, Guangzhou 510630, China

## Abstract

**Aims:**

To investigate the distribution of diabetic retinopathy (DR) by sex in patients with type 2 diabetes mellitus (T2DM) in a twelve-province cross-sectional study in China.

**Methods:**

Patients with T2DM, whose ages were ≥18 years, were recruited from 76 cities/counties in 12 provinces in mainland China between January 2015 and December 2018. All participants received a standardized interview, eye examinations, and digital fundus photography. The presence and severity of DR were diagnosed and classified by retina specialists according to the DR domestic typing method.

**Results:**

A total of 12,766 participants (5963 males and 6803 females) were eligible for this study. The total prevalence of DR was 30.1%. Females exhibited a significantly higher prevalence of DR than males (31.1% vs. 29.0%, *P* = 0.011). A multivariate logistic regression analysis confirmed that female sex was an independent predictor for a higher prevalence of DR after adjusting for age, the duration of diabetes, economic status, and the presence of hypertension (OR: 1.096, 95% CI: 1.013-1.186, *P* = 0.023). Even after stratification by the diabetic duration, age, and economic status, female sex was still independently associated with the presence of DR in patients whose T2DM history was more than 10 years, whose ages were over 60 years, or who were in a relatively intermediate economic area.

**Conclusion:**

Females had a higher prevalence of DR than males in T2DM patients with a diabetic history of more than 10 years, ages over 60 years, or a relatively intermediate economic status.

## 1. Introduction

Type 2 diabetes mellitus (T2DM) is highly prevalent worldwide and is increasing rapidly [1]. Diabetic retinopathy (DR) is one of the most frequent and serious microvascular complications in T2DM. The overall prevalence of DR is estimated to be 34.6% [2] and ranges from 11.9% to 43.1% in mainland China [3, 4]. DR remains a leading cause of blindness among working age populations in both developed and developing countries [5]. However, limited treatment options are available for DR when it progresses to late-stage disease, in which vision is already impaired. Late-stage DR requires repeated treatments (laser photocoagulation or intravitreal injections of antivascular endothelial growth factor agents) with unsatisfactory effects, which result in a high socioeconomic burden [6–8]. Therefore, it is valuable to identify the risk factors that will help to prevent the occurrence and delay the progression of DR.

Multiple risk factors contribute to DR, such as chronic hyperglycemia, hypertension, dyslipidemia, a long duration of diabetes, overweight, and age [9, 10]. It remains ambiguous which sex is more susceptible to DR. A study of 120,000 cases from Germany and Australia shows that females are more likely to suffer from DR than are males [11]. Similarly, studies from Britain and Japan show that females are more prone to suffer from visual impairment than are males [12, 13]. However, there are some studies from the United States and India that indicate that male sex is a risk factor for DR [14–16]. In particular, the United Kingdom Prospective Diabetic Study, which is a milestone T2DM study, presented that the progression of retinopathy was associated with male sex [17]. This inconsistency highlights the need for further investigation of the association between DR and sex. Furthermore, there is a lack of data about the Chinese population, which has the largest number of T2DM patients in the world [1]. Therefore, the primary objective of this study was to explore the relationship between sex and DR in T2DM patients; the secondary purpose was to determine the prevalence of DR in mainland China.

## 2. Materials and Methods

### 2.1. Study Population and Data Source

This cross-sectional study was a population-based study. Participants were enrolled from 12 provinces, including 76 cities/counties and 381 community health service centers and hospitals in mainland China, between January 2015 and December 2018. The protocol was approved by the Ethics Committee of the Third Affiliated Hospital of Sun Yat-sen University. All participants signed an informed written consent form.

### 2.2. Study Design

**Figure 1:**
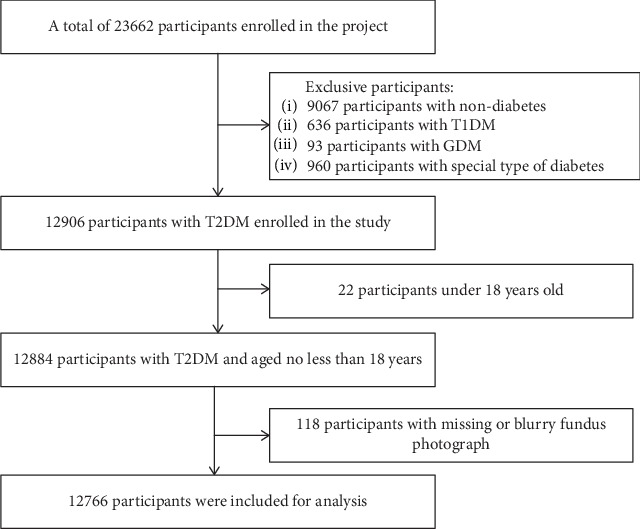
Flowchart of the study.

A total of 23,662 participants with T2DM who were at least 18 years old were enrolled in this cross-sectional study. T2DM was diagnosed according to the 1999 criteria of the World Health Organization (WHO) [18]. Twelve thousand eight hundred eighty-four patients were eligible. Patients with serious mental illness and other situations in which the requirements of the agreement cannot be complied with (e.g., patients unable to take care of themselves, alcoholics, and drug abuser) were excluded from the study, as were pregnant and lactating women. One hundred eighteen subjects with missing data or blurry fundus photographs were excluded. A total of 12,766 subjects were finally included in the analysis (Figure 1).

### 2.3. Eye Examination

Eye examinations were performed on all participants according to standard operation procedure by specific trained ophthalmologists. The eye examinations included visual acuity measurements, tonometry, and an anterior ocular structure and fundus examination using a standard protocol. Intraocular pressure in both eyes was measured with a noncontact tonometer (VISUPLAN 500 Non-Contact Tonometer, Carl Zeiss Vision Inc., San Diego, USA). The external and anterior ocular segment was examined by slit lamp biomicroscopy (BQ900; Haag-Streit, Bern, Switzerland). Two 45° field digital, colored, nonstereoscopic fundal photographs of each eye were taken in the macula-centered and posterior pole by a nonmydriatic auto fundus camera (Canon CR-DGi retinal camera; Canon, Tokyo, Japan, or TRC-NW400 Non-Mydriatic Retinal Camera, Topcon, Tokyo, Japan).

### 2.4. Assessment of DR

DR was diagnosed and graded based on fundus photographs according to the guidelines of 1985 and classified into two types (nonproliferative retinopathy and proliferative retinopathy) and six stages, as shown in Supplemental Table 1. Stages 1-3 apply to nonproliferative retinopathy, and stages 4-6 apply to proliferative retinopathy. Details of these guidelines are shown in Supplemental Table 1.

### 2.5. Data Collection

A standardized questionnaire was applied to collect basic information, such as age, sex, previous eye disease history and eye surgery history, comorbidities, the duration of diabetes, and region. The patients' chief complaint was also recorded by trained doctors. All of physicians and ophthalmologists in this study were trained before research. The economic level variable was categorized into tertiles according to the 2018 per capita gross regional product data from the National Bureau of Statistics for each city, as shown in Fig S1. Hypertension is defined as a blood pressure ≥ 140 mmHg systolic or ≥90 mmHg diastolic or current use of antihypertensive medication according to the European Society of Hypertension (ESH) and the European Society of Cardiology (ESC) [19].

### 2.6. Statistical Analyses

Database management and statistical analysis were performed using PASW 22.0 for Windows (IBM Inc., Armonk, USA). Descriptive statistics are presented as the mean (standard deviation) or median (interquartile range) for continuous variables and as numbers (percentages) for categorical variables. Continuous variables were compared by *t*-tests, while categorical variables were compared by Pearson chi-squared tests to determine between sex differences. Univariate logistic regression analysis was performed to assess the nonadjusted relationships between sex and the prevalence of DR. Odds ratios (ORs) and 95% confidence intervals (CIs) were estimated for the association between DR and sex using males as the reference group. After that estimation, age, the duration of diabetes, the presence of hypertension, and the economic level were adjusted in the multivariate logistic regression analysis models. To determine whether the presence of hypertension, the duration of diabetes, age, and economic levels affect the relationship between sex and the prevalence of DR, subgroup analyses were performed based on the presence of hypertension (without and with), the duration of diabetes (<10 years and ≥10 years), age (<60 years old and ≥60 years old), and economic level tertiles. A two-tailed *P* < 0.05 was considered statistically significant.

## 3. Results

### 3.1. Participants' Characteristics

A total of 12,766 participants (5963 males and 6803 females; mean age of 61.9 ± 11.5 years) were eligible for this study. The median duration of diabetes was 5.0 (2.0-10.0) years. There were 6434 (50.4%) patients who had both T2DM and hypertension. The distribution of economic levels among the participants is shown in Table 1. A total of 50.1% of the participants came from intermediate economic areas (Guangdong, Zhejiang, Fujian, Shandong, and Inner Mongolia), 40.3% came from low economic areas (Sichuan, Gansu, Hebei, Shanxi, Jiangxi, Yunnan, Shanxi, Henan, Hubei, Hunan, and Guizhou), and 9.6% came from relatively high economic areas (Beijing, Jiangsu). The characteristics of the participants by sex are shown in Table 1. Compared with males with T2DM, the female population was older (63.3 ± 10.7 vs. 60.2 ± 12.1, *P* < 0.001), had a longer duration of diabetes (6.0 (2.0-10.0) vs. 5.0 (2.0-10.0), *P* < 0.001), and had a higher prevalence of hypertension (54.8% vs. 45.4%, *P* < 0.001).

### 3.2. The Prevalence of Diabetic Retinopathy

In the present study, 3847 patients (30.1%) suffered from DR. The females exhibited a significantly higher prevalence of DR than the males (31.1% vs. 29.0%, *P* = 0.011) (Table 2). No significant difference was observed in the severity of DR between the females and the males.

### 3.3. Univariate and Multivariate Logistic Regression Analysis

As shown in Table 3, female sex was found to be potentially correlated with the presence of DR (*P* < 0.05). The multivariate logistic regression analysis confirmed that female sex was an independent predictor for a higher prevalence of DR after adjusting for age, the duration of diabetes, economic levels, and the presence of hypertension (OR: 1.096, 95% CI: 1.013-1.186, *P* = 0.023).

### 3.4. Subgroup Analysis

**Figure 2:**
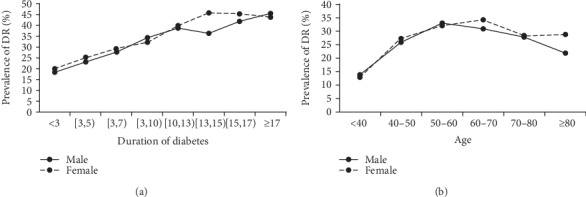
The prevalence of DR in gender difference in subgroup analysis. (a) The trend of prevalence of DR in gender difference with the duration of diabetes. (b) The trend of prevalence of DR in gender difference with age.

To preclude the influence of hypertension, the duration of diabetes, age, and economic levels, these four factors were introduced in the subgroup analyses. As shown in Figures 2(a) and 2(b), a remarkable sex difference was observed in patients whose diabetes duration was more than 10 years and in patients who were older than 60 years old. The subgroup logistic regression analysis, which was stratified by the presence of hypertension (without vs. with), the duration of diabetes (<10 years vs. ≥10 years), age (<60 years old vs. ≥60 years old), and economic levels (tertile of per capita gross regional product), was further performed (Table 4). Compared with the male participants, the female participants had a significantly higher prevalence of DR in the diabetic duration ≥ 10 years subgroup (OR: 1.150, 95% CI: 1.012-1.306, *P* = 0.032), the age ≥ 60 years subgroup (OR: 1.141, 95% CI: 1.031-1.262, *P* = 0.010), and intermediate economic area subgroup (OR: 1.123, 95% CI: 1.001-1.259, *P* = 0.048). No sex difference was found in the hypertension, age < 60 years, diabetic duration < 10 years, or high or low economic area subgroups (*P* > 0.05).

## 4. Discussion

This national cross-sectional study with a large sample size showed that the overall prevalence of DR was 30.1% in mainland China. Moreover, the study also indicated that the female participants with T2DM exhibited a higher prevalence of DR than the male participants, particularly for the subjects with a diabetic history of more than 10 years, who were over 60 years old, or who were from areas of a relatively intermediate economic level. This result suggested that female sex was an independent risk factor for DR in T2DM patients.

The prevalence of DR (30.1%) in our study was similar to that in the studies from the United States (33.2%) [20] and Singapore (30.4%-35.0%) [21, 22]. The prevalence of DR in previous studies in Chinese ranged from 11.9% to 43.1% [3, 4]. This discrepancy may be due to study design, DR grading standards, and regional differences. The prevalence of 11.9% came from the general population in the northeast area of China with nonmydriatic retinal photographs [3], whereas the percentage of 43.1% was from a rural population in northern China with retinal photographs obtained after pupil dilation [4]. These two results suggested that DR prevalence varied by region, population, and retinal measurement. A recent multihospital-based population study across China with 16,305 participants showed that the overall age- and sex-standardized prevalence of DR was 27.9% [23], which was similar to our study. The participants in our study came from 76 cities/counties in 12 provinces, including the northern, southern, central, eastern, and western regions of mainland China, which enhanced the results.

The association between age and DR is still controversial. Some previous studies showed that older onset patients with diabetes had a higher prevalence of DR, and the prevalence of DR increased with age at diagnosis [23]. This may be attributed to fact that older patients had longer diabetes duration, which was a strong risk factor for the prevalence of DR [2, 3, 10, 15, 23]. Our study showed that the prevalence of DR increased with age in the age groups below 60 years but decreased with age in both men and women in the groups above 60 years. Consistent with our study, previous study showed that patients were getting less likely to suffer from DR every 10 years after 60 years of age [24]. Moreover, some studies showed that patients with young onset diabetes had a higher prevalence of DR [25–27], even for patients with similar diabetes duration [25]. There are several possible reasons for our result. Firstly, there is an eye problem with reduced vision gradient by age, such as hyperopia. Elderly people tend to consider the vision loss problem caused by ageing, thus would be less motivated for eye checking than younger patients. In addition, the elderly T2DM patients are prone to cataract, which might reduce the detection of DR under nonmydriatic fundus photography. The elderly patients with DR had the likelihood of suffering from other severe chronic complications, such as cardiovascular disease, which made them pay less attention to the eye problem or less motivated to access community health service centers and hospitals for the eye problem. Moreover, DR have higher cardiovascular disease and all-cause mortality in older DR populations [28, 29], which might lead to survival bias. Therefore, the proportion of DR has decreased after 60 years of age in both men and women in our study.

Accumulating evidence indicates that gender appears to be a significant factor in DR [10, 13, 30]. However, it is still debatable which sex is more prone to DR. Some studies have indicated that female sex was an independent risk factor for the incidence and development of overall DR and proliferation DR (PDR) [11–13]. Some studies have shown that the presence and severity of DR are more strongly associated with male sex [10, 14–17]. Other studies suggested that there was no discernible sex difference in the prevalence of DR [2, 31]. This discrepancy may be due to the differences in study designs, patient characteristics (such as diabetes duration and comorbidity), and characteristics of populations sampled (such as race, region, and economic level), which influence DR [2, 10–17, 31]. Interestingly, our study found that sex differences only existed in the T2DM patients who had a longer diabetic duration (≥10 years), who were over 60 years old, or who were from an intermediate economic area. Taken together, these inconsistent results suggest that further investigation into the relationship between sex and DR is required.

The mechanisms by which female sex contributes to the prevalence of DR in T2DM patients are still unknown. There are some potential explanations. First, the outcome may be due to estrogen. A meta-analysis showed that the prevalence of DR in DM patients peaked between the ages of 60 and 69 [32], at which point females were postmenopausal. Our study found that only females over the age of 60 years had a higher prevalence of DR than males. This sex difference was not present in those who were under 60 years of age. These results suggest that estrogen protects the occurrence and development of DR. Indeed, 17*β*-estradiol (E2) was found to protect RGC-5 cells from high-glucose-induced damage via the mitochondrial pathway [33]. In addition, estrogen was found to be an important regulator of blood flow in the retina and plays a protective role by decreasing vascular resistance in large ocular vessels [34]. A previous study also indicated that hormone therapy was beneficial for ocular vascular disease in postmenopausal females [35]. This finding may be the reason why postmenopausal females with T2DM were more susceptible to DR. Furthermore, estrogen has a protective effect on the occurrence of T2DM and improves its treatment [36–38]. Previous studies showed that the prevalence of DR increased steeply with the duration of DM [32]. As a result, estrogen can benefit DR by preventing the occurrence of T2DM and delaying its progression. Our results were consistent with those of previous studies and further found that females with T2DM and long-term T2DM durations have an elevated risk of DR. Taken together, estrogen benefits DR in many ways. Further studies are required to determine the underlying mechanism by which estrogen influences DR. Second, a recent systematic review reported that lower social economic levels and old age were attributed to diabetic complications [39]. In the present study, sex differences in the prevalence of DR were observed, particularly for those who came from the relatively intermediate economic areas. As a result, the sex difference in our study may be due to the imbalanced distribution of risk factors caused by differences in the social economic levels. There are some socioeconomic factors related to DR prevalence by gender. Among socioeconomic factors, a lower education level was associated with higher DR risk [40]. There is a common health gradient by education. People with lower education levels have weak awareness of self-care and have the higher probability of poor health [41], which affects their health and may increase the risk of DR. Additionally, like those with lower education levels, people with low household income do not have enough healthcare, especially among women. Household income strongly affects the health of elderly women. A higher income to some extent indicates having a healthy lifestyle, more physical exercise, and better access to healthcare services [42]. For low socioeconomic status, women are prone to obesity compared with men, as there are differences in nutritional consumption and stress depending on the level of socioeconomic status, especially for older women, as the 2010-2012 China National Nutrition and Health Survey (CNNHS) showing that women aged 60 years and older had a higher overweight/obesity prevalence than men [43]. And a meta-analysis showed that obesity was a risk factor for DR [44]. Therefore, the gender difference in social-economic factors, such as education, household income, and self-care awareness, may partly lead to the gender difference in the DR risk. Notably, there was no sex difference in the DR patients whose ages were younger than 60 years, where estrogen could protect females from DR. This result suggests that there may be other factors that influence DR, which is worth of further study. Additional research may provide a new potential target for DR prevention and treatment.

There are some factors that limit the extent to which our results can be generalized. First, our sampling methods were not strictly stratified, resulting in insufficient representation. Second, a regional selection bias could not be excluded in this study. The subjects in this study were mainly from northern and southern China. For a more comprehensive understanding of the prevalence of DR in mainland China, nationwide, population-based research studies are needed. Another limitation is that the detailed clinical characteristics are relatively insufficient. As a result, we were not able to analyze more profoundly to find more potential influential factors. In addition, we did not adjust for the potential confounders completely. A large sample size and nationwide enrollment from 76 cities in 12 provinces are the strength of our study, which will add important information about DR in Chinese T2DM patients.

In summary, our study demonstrated that the prevalence of DR in T2DM was 30.1% in mainland China and that female sex was independently associated with the prevalence of DR, particularly for T2DM patients over 60 years of age, who had a diabetic duration of more than 10 years, or who lived in relatively intermediate economic regions. This group of T2DM patients should receive more concern in the clinic, and the underlying mechanism of the female as a risk factor for DR is worth of further study, which may provide a new potential target for DR prevention and treatment.

## Figures and Tables

**Table 1:** Characteristics of participants.

	Total (*N* = 12766)	Male (*N* = 5963)	Female (*N* = 6803)	*P* value
Age (year)	61.9 (11.5)	60.2 (12.1)	63.3 (10.7)	<0.001
Duration of diabetes (years)	5.0 (2.0-10.0)	5.0 (2.0-10.0)	6.0 (2.0-10.0)	<0.001
Hypertension, *n* (%)	6434 (50.4)	2708 (45.4)	3726 (54.8)	<0.001
Regional economic level, *n* (%)				<0.001
T1 (Sichuan, Gansu, Hebei, Shanxi, Jiangxi, Yunnan, Shaanxi, Henan, Hubei, Hunan, and Guizhou)	5139 (40.3)	2237 (37.5)	2902 (42.7)	
T2 (Guangdong, Zhejiang, Fujian, Shandong, and Inner Mongolia)	6399 (50.1)	3112 (52.2)	3287 (48.3)	
T3 (Beijing, Jiangsu)	1228 (9.6)	614 (10.3)	614 (9.0)	

Data are the mean (standard deviation), median (25th to 75th percentiles), or *n* (%); the cities were tertiled by the regional economic level according to the data of 2018 per capita gross regional product from the National Bureau of Statistics. T1 referred to 31,336.13-67,627.83, T2 referred to 67,627.83-103,919.54, and T3 referred to 103,919.54-140,211.24.

**Table 2:** Prevalence and severity of diabetic retinopathy of participants with gender difference.

	Total (*N* = 12,766)	Male (*N* = 5963)	Female (*N* = 6803)	*P* value
Diabetic retinopathy, *n* (%)	3847 (30.1)	1731 (29.0)	2116 (31.1)	0.011
Nonproliferative, *n* (%)				
Stage I	1822 (14.3)	843 (14.1)	981 (14.5)	0.141
Stage II	1426 (11.2)	634 (10.8)	791 (11.6)	
Stage III	416 (3.3)	168 (2.8)	247 (3.6)	
Proliferative, *n* (%)				
Stage IV	137 (1.1)	61 (1.0)	76 (1.1)	
Stage V	27 (0.2)	13 (0.2)	14 (0.2)	
Stage VI	19 (0.1)	12 (0.2)	7 (0.1)	

Data are *n* (%).

**Table 3:** Logistic regression analysis assessing the relationships of gender with diabetic retinopathy.

	Univariate analysis			Multivariate analysis		
	OR	95% CI	*P* value	OR	95% CI	*P* value
Male	1	—	—	—	—	—
Female	1.104	1.023-1.191	0.011	1.096	1.013-1.186	0.023
Hypertension				1.215	1.120-1.318	<0.001
Age				0.993	0.990-0.997	0.001
Duration of diabetes				1.069	1.062-1.075	<0.001
Regional economic level—T1				1	—	—
Regional economic level—T2				0.712	0.618-0.819	<0.001
Regional economic level—T3				0.730	0.673-0.792	<0.001

Results are given as odds ratios and 95% confidence intervals (OR; 95% CI). The subjects were divided into nondiabetic retinopathy (DR) and DR. Gender was analyzed in the univariate logistic regression analysis, using male as the reference group. Then, a multivariate logistic regression analysis was adjusted for age, duration of diabetes, hypertension, and regional economic level.

**Table 4:** Subgroup logistic regression analysis assessing the relationships of gender with diabetic retinopathy.

	Univariate analysis			Multivariate analysis		
	OR	95% CI	*P* value	OR	95% CI	*P* value
*Stratified by hypertension*						
Male	1	—	—	1	—	—
Female						
Without hypertension	1.143	1.028-1.127	0.014	1.094	0.980-1.222	0.109
With hypertension	1.096	0.982-1.223	0.102	1.091	0.974-1.222	0.131
*Stratified by duration of diabetes*						
Male	1	—	—	1	—	—
Female						
<10 years	1.090	0.987-1.203	0.090	1.057	0.955-1.170	0.284
≥10 years	1.069	0.944-1.211	0.291	1.150	1.012-1.306	0.032
*Stratified by age*						
Male	1	—	—	1	—	—
Female						
<60 years old	1.057	0.936-1.195	0.373	0.986	0.867-1.122	0.830
≥60 years old	1.122	1.017-1.238	0.022	1.141	1.031-1.262	0.010
*Stratified by regional economic level*						
Male	1	—	—	1	—	—
Female						
T1 (31,336.13-67,627.83)	1.051	0.935-1.182	0.403	1.054	0.934-1.188	0.396
T2 (67,627.83-103,919.54)	1.135	1.017-1.267	0.024	1.123	1.001-1.259	0.048
T3 (103,919.54-140,211.24)	1.040	0.814-1.329	0.754	1.148	0.889-1.481	0.290

Results are given as odds ratios and 95% confidence intervals (OR; 95% CI). The subjects were divided into nondiabetic retinopathy (DR) and DR. Gender was analyzed in the univariate logistic regression analysis in T2DM participants stratified by hypertension, duration of diabetes, age, and regional economic level, using male as the reference group. Then, a multivariate logistic regression analysis in T2DM participants stratified by hypertension was adjusted for age, duration of diabetes, and regional economic level; a multivariate logistic regression analysis in T2DM participants stratified by duration of diabetes was adjusted for age, duration of diabetes, hypertension, and regional economic level; a multivariate logistic regression analysis in T2DM participants stratified by age was adjusted for age, duration of diabetes, hypertension, and regional economic level. A multivariate logistic regression analysis in T2DM participants stratified by the regional economic level was adjusted for age, duration of diabetes, and hypertension.

## Data Availability

The data used to support the findings of this study are available from the corresponding author upon request.

## Supplementary Materials

Supplementary Materials Supplemental Table 1: classification of diabetic retinopathy. Diabetic retinopathy domestic typing (1985). Fig S1: per capita gross regional product of 2018 in China. Data source: National Bureau of Statistics.
