# Supplementary material for: Females with Type 2 Diabetes Mellitus Are Prone to Diabetic Retinopathy: A Twelve-Province Cross-Sectional Study in China
Source: J Diabetes Res. 2020 Apr 21;2020:5814296. doi: 10.1155/2020/5814296 (PMC7191394; doi:10.1155/2020/5814296)

Supplemental Table 1: Classification of diabetic retinopathy. Diabetic retinopathy domestic typing (1985)

Type	Stage	Retinopathy
Non-proliferative retinopathy	I	microaneurysms or combined with small blood loss
	II	stage I + hard exudates
	III	stage II + cotton wool spots
Proliferative retinopathy	IV	neovascularization in addition to the above lesions
	V	neovascularization and proliferative membranes form
	VI	stage V with retinal detachment

Supplementary figure legend:

Fig. S1：Per capita gross regional product of 2018 in China. Data source: National Bureau of Statistics.

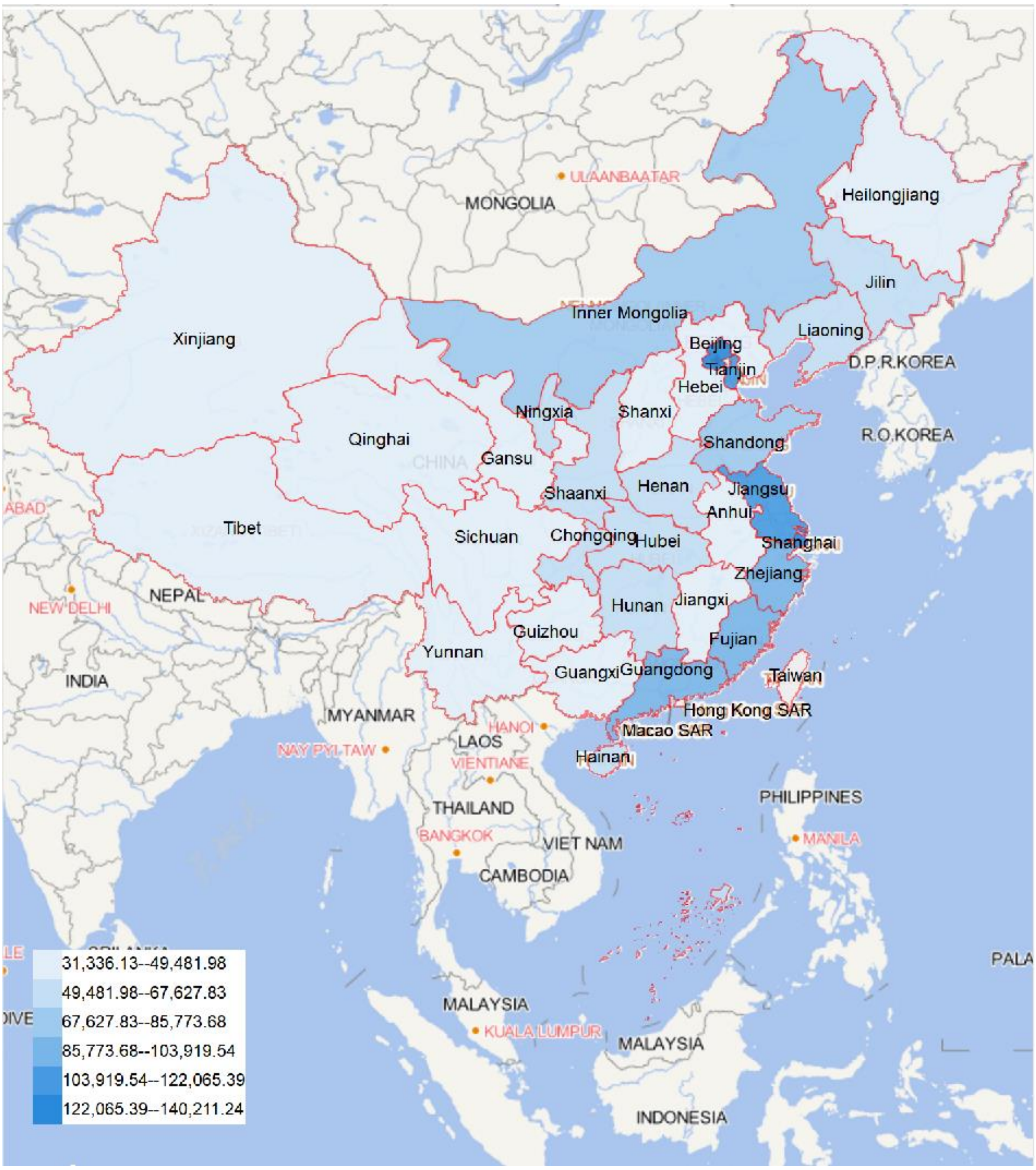

Supplement: Supplementary Materials — Supplemental Table 1: classification of diabetic retinopathy. Diabetic retinopathy domestic typing (1985). Fig S1: per capita gross regional product of 2018 in China. Data source: National Bureau of Statistics. [file 5814296.f1.pdf]
